# Engagement, Predictors, and Outcomes of a Trauma Recovery Digital Mental Health Intervention: Longitudinal Study

**DOI:** 10.2196/35048

**Published:** 2022-05-02

**Authors:** Carolyn M Yeager, Charles C Benight

**Affiliations:** 1 Lyda Hill Institute for Human Resilience University of Colorado Colorado Springs Colorado Springs, CO United States

**Keywords:** engagement, digital health, digital mental health intervention, social cognitive theory, SCT, self-efficacy, outcome expectations, trauma, posttraumatic stress disorder, PTSD

## Abstract

**Background:**

Worldwide, exposure to potentially traumatic events is extremely common, and many individuals develop posttraumatic stress disorder (PTSD) along with other disorders. Unfortunately, considerable barriers to treatment exist. A promising approach to overcoming treatment barriers is a digital mental health intervention (DMHI). However, engagement with DMHIs is a concern, and theoretically based research in this area is sparse and often inconclusive.

**Objective:**

The focus of this study is on the complex issue of DMHI engagement. On the basis of the social cognitive theory framework, the conceptualization of engagement and a theoretically based model of predictors and outcomes were investigated using a DMHI for trauma recovery.

**Methods:**

A 6-week longitudinal study with a national sample of survivors of trauma was conducted to measure engagement, predictors of engagement, and mediational pathways to symptom reduction while using a trauma recovery DMHI (time 1: N=915; time 2: N=350; time 3: N=168; and time 4: N=101).

**Results:**

Confirmatory factor analysis of the engagement latent constructs of duration, frequency, interest, attention, and affect produced an acceptable model fit (*χ^2^*_2_=8.3; *P*=.02; comparative fit index 0.973; root mean square error of approximation 0.059; 90% CI 0.022-0.103). Using the latent construct, the longitudinal theoretical model demonstrated adequate model fit (comparative fit index 0.929; root mean square error of approximation 0.052; 90% CI 0.040-0.064), indicating that engagement self-efficacy (β=.35; *P*<.001) and outcome expectations (β=.37; *P*<.001) were significant predictors of engagement (*R*^2^=39%). The overall indirect effect between engagement and PTSD symptom reduction was significant (β=–.065; *P<*.001; 90% CI –0.071 to –0.058). This relationship was serially mediated by both skill activation self-efficacy (β=.80; *P*<.001) and trauma coping self-efficacy (β=.40; *P*<.001), which predicted a reduction in PTSD symptoms (β=−.20; *P*=.02).

**Conclusions:**

The results of this study may provide a solid foundation for formalizing the nascent science of engagement. Engagement conceptualization comprised general measures of attention, interest, affect, and use that could be applied to other applications. The longitudinal research model supported 2 theoretically based predictors of engagement: engagement self-efficacy and outcome expectancies. A total of 2 task-specific self-efficacies—skill activation and trauma coping—proved to be significant mediators between engagement and symptom reduction. Taken together, this model can be applied to other DMHIs to understand engagement, as well as predictors and mechanisms of action. Ultimately, this could help improve the design and development of engaging and effective trauma recovery DMHIs.

## Introduction

### Background

The World Mental Health Survey Consortium indicated that >70% of adults are exposed to traumatic events [1]. In the United States, approximately 90% of people are estimated to have at least one exposure to a traumatic event during their lifetime [2]. The COVID-19 pandemic has presented additional challenges resulting in an increased global demand for mental health services, along with increases in trauma exposure in its aftermath [3]. As trauma exposure increases, so do the risks of developing posttraumatic stress disorder (PTSD), along with other mental and physical health conditions [4]. Costs associated with mental health disorders are significant, accounting for approximately 7% of the global burden of disease and 19% of all years lived with disability [5]. Despite its high prevalence and societal costs, treatment coverage remains poor, resulting in a global mental health treatment gap [6]. Substantial barriers to mental health treatment include perceived stigma, access, costs, and lack of trained personnel. Rural and underserved communities are especially vulnerable to these barriers, which have been exacerbated in the wake of the COVID-19 pandemic [7].

### Digital Mental Health Interventions

A promising approach to overcoming these barriers is the use of technology to reach more people at a low cost in a structured and confidential format [8,9]. Technology to promote mental health and behavior change is referred to as a digital mental health intervention (DMHI). The acceptance of DMHI apps continues to increase, and downloads have risen exponentially since the proliferation of the COVID-19 pandemic [10]. Several systematic reviews have concluded that although DMHIs are growing in popularity, evidence of their efficacy is still limited [10,11]. Research suggests that some inconclusive findings on DMHI effectiveness may be related to a lack of engagement [12]. As engagement may influence intervention outcomes, a greater understanding of engagement and the factors that influence DMHI engagement is essential [13].

However, despite its importance, a consistent engagement conceptualization is lacking [14]. The term *engagement*, broadly defined as attention, interest, and use of a DMHI [15], has been used in many ways, yielding inconsistent findings and making it challenging to synthesize reliable models and measures. The lack of guidelines or specificity makes it difficult to measure, interpret, and compare the engagement metrics across DMHIs. A recent systematic review concluded that the field of DMHIs depends on user engagement, and the lack of clear definitions and standards can be harmful to the field [16].

This study sought to address this gap by extending previous engagement research [17] to examine a theoretical framework for the conceptualization of engagement, predictors of engagement, and the relationship between engagement and outcomes. Before presenting the model, the engagement conceptualization is described.

### Engagement

Most studies agree that engagement includes some interaction with a DMHI [18]; however, there is little agreement as to what exactly engagement is, its bounds, and a precise conceptualization of the concept in general (see Yeager and Benight [19] for a full review). Systematic reviews of engagement research concluded that the definition of engagement must go beyond objective measures of use to include subjective measures of attention, interest, and affect [14-16]. This definition aligns with the social cognitive theory (SCT) framework [20], where observed behaviors (ie, DMHI use), cognitive factors (ie, attention and interest), and personal factors (ie, affect) interact to define the more complex process of engagement. As far as we are aware, this is one of the first studies to explore a multidimensional conceptualization of engagement that includes subjective measures of attention, interest, and affect and objective measures of use.

### Longitudinal Engagement Research Model

#### Overview

**Figure 1:**
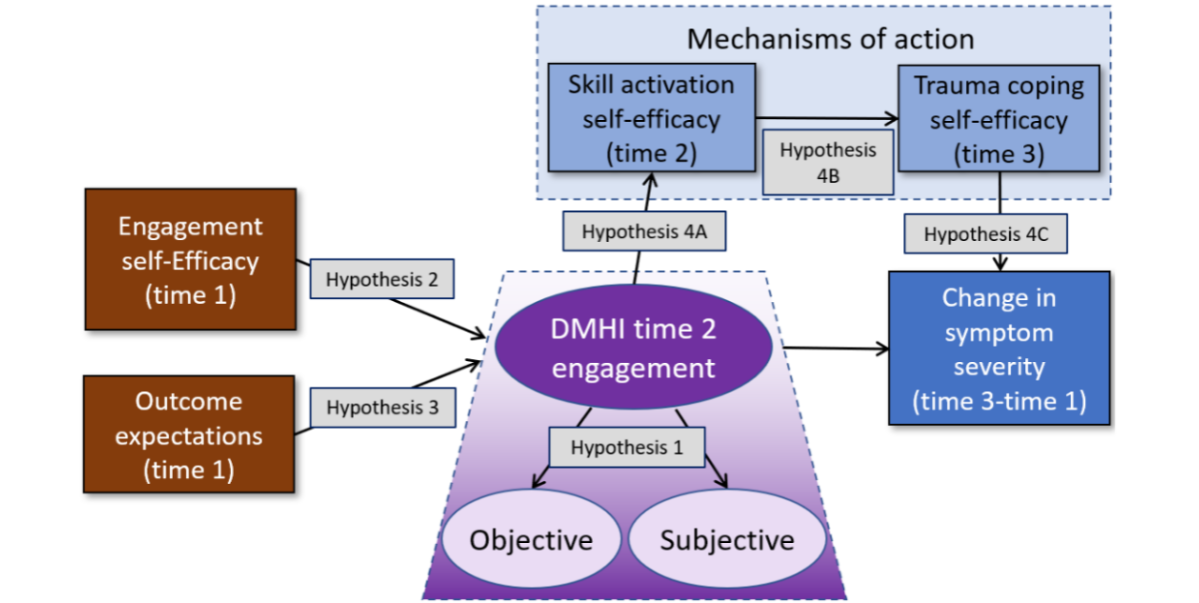
Proposed longitudinal digital mental health intervention engagement model. Predictors of engagement are shown in brown, objective and subjective measures of engagement are shown in purple, and the direct and indirect relationships to digital mental health intervention outcomes are shown in blue. Hypotheses are also indicated. DMHI: digital mental health intervention.

On the basis of this engagement conceptualization, a longitudinal research model was developed and tested (Figure 1). This model was built on several frameworks [15,17,21] and included predictors of engagement (shown in brown), objective and subjective measures of engagement (shown in purple), and direct and indirect relationships to DMHI outcomes (shown in blue).

#### Engagement Predictors

Predictors of engagement can include countless combinations of user characteristics and DMHI design components [22,23]. Using a theoretical framework is essential to begin to whittle down the most important factors [12]. SCT, which offers a parsimonious, self-regulatory framework for motivating DMHI engagement [20], suggests that behaviors are performed if one perceives confidence in one’s ability, there are few external barriers, and the behavior is worth the effort. Self-efficacy [24] and outcome expectations [25] are key constructs in this self-regulatory process. SCT suggests that predictors of engagement include both personal (eg, appraisals) and external (eg, DMHI characteristics) factors.

Engagement self-efficacy reflects appraisals of one’s ability to initiate and maintain engagement with a DMHI despite barriers associated with using a DMHI [26] that focuses on trauma recovery [27]. Engagement self-efficacy incorporates both confidence in using technology and confidence in addressing traumatic stress symptoms. Individuals high in engagement self-efficacy imagine success in using a DMHI and are more likely to initiate a new behavior, invest more effort, and persist longer than those who are less self-efficacious [28]. These individuals may persist despite challenges associated with using technology and avoidant behaviors, a hallmark symptom cluster of PTSD [29]. Therefore, engagement self-efficacy is predicted to have a positive effect on engagement.

Outcome expectations are the estimation that a given behavior, once performed, will lead to desired outcomes [30]. Outcome expectancy includes DMHI characteristics such as beliefs that the DMHI will be effective [31]. For this study, outcome expectations are defined as perceptions that using the DMHI will increase one’s ability to cope with symptoms associated with their trauma. Low outcome expectations are often cited as barriers to in-person evidence-based treatment for those with PTSD [32]. Therefore, higher outcome expectations are predicted to have a positive effect on DMHI engagement.

#### Engagement Outcomes

Understanding the full picture of DMHI effectiveness must also include the anticipated effects of engagement on important postintervention outcomes. The ambiguity demonstrated in the predictors of engagement research was also found in research examining the relationship between engagement and DMHI outcomes [33,34]. Research has shown that there is a dose-response relationship: the greater the use, the greater the positive effects [17,35]. However, not all DMHIs show this relationship [31,36,37], which may be attributable to several factors, including a lack of engagement consensus and lack of consideration of DMHIs’ mechanisms of action [34].

These mechanisms can serve as mediators between DMHI engagement and desired outcomes. Perski et al [15] found that mechanisms of action include beliefs, knowledge, motivation, self-efficacy, and skill practice. To increase our understanding of these potential mechanisms, our model included 2 serial mediators between engagement and outcomes (Figure 1).

On the basis of the SCT, we examined 2 forms of self-efficacy, measured at subsequent periods, as potential mechanisms of action. According to Bandura [20], self-efficacy is a context-specific assessment of competence in performing a specific task. These different types of task-specific self-efficacy, which is an assessment targeting distinct appraisals across different groups of behaviors, include skill activation and coping with trauma symptoms.

Skill activation self-efficacy refers to a person’s confidence in performing the specific skills taught by the DMHI, and coping self-efficacy for trauma (CSE-T) refers to a person’s confidence in managing their internal and external posttraumatic recovery demands [27]. In our theorizing, greater engagement with the DMHI enhanced skill activation self-efficacy, which led to subsequent increases in CSE-T. In the proposed model, skill activation self-efficacy was measured after using the DMHI for approximately 1 week (time 2 [T2]), and CSE-T was measured after using the DHMI for approximately 2 weeks (time 3 [T3]).

Skill activation self-efficacy reflects confidence in one’s ability to use the new skills learned from the DMHI, despite associated barriers. According to Bandura [20], confidence in practicing DMHI skills precedes the actual use of the skill, and this step is often ignored in the literature. The specific trauma recovery skills taught by the DHMI are relaxation, increasing social support, managing triggers, identifying unhelpful thoughts, and using healthy coping strategies. Practicing these skills may prove to be more challenging to adhere to than expected; however, a self-efficacious person will respond more confidently, with better strategies, effort, and persistence in overcoming such hurdles. We hypothesized that as a person engages more with the DMHI, their confidence in using DMHI skills will also increase.

Those confident in practicing the DHMI trauma recovery skills may also increase their confidence in managing posttraumatic recovery demands. Research has shown that the aforementioned skills can improve one’s ability to manage trauma-related symptoms [38]. Consequently, increasing confidence in performing DMHI skills (ie, skill activation self-efficacy) was hypothesized to increase one’s perceived ability to cope with trauma-related symptoms (ie, CSE-T). Those with high CSE-T perceive that they have the necessary coping strategies to manage posttraumatic recovery demands and, hence, experience fewer psychological symptoms [38]. A recent longitudinal study showed that participants who used the DMHI for 3 weeks experienced an increase in CSE-T and clinically lower PTSD symptoms [39]. Therefore, CSE-T was included as a mediator and predicted to have a significant and negative effect on PTSD symptoms (ie, increases in CSE-T result in decreases in symptom severity).

From the engagement conceptualization and longitudinal theoretical models, several hypotheses were put forth (Textbox 1 and Figure 1).

**Hypotheses of the study. CSE-T: coping self-efficacy for trauma.**

**Study hypotheses**

- Hypothesis 1: a relationship exists between the observed subjective and objective engagement variables and their underlying engagement latent construct, as demonstrated by the adequate model fit of the latent construct. The engagement latent construct included objective measures of use and subjective measures of attention, interest, and affect.
- Hypothesis 2: participants with higher engagement self-efficacy would experience higher levels of engagement.
- Hypothesis 3: participants with higher outcome expectations would experience higher levels of engagement.
- Hypothesis 4: skill activation self-efficacy and coping self-efficacy for trauma would serially mediate the relationship between engagement and outcome:
  - Hypothesis 4A: participants with higher levels of engagement would experience higher levels of skill activation self-efficacy.
  - Hypothesis 4B: participants with higher levels of skill activation self-efficacy would experience higher levels of CSE-T.
  - Hypothesis 4C: participants with higher CSE-T would experience a greater reduction in trauma symptoms.

## Methods

### Overview

A 6-week correlational, longitudinal study was performed using a DMHI with a population of survivors of trauma to test the proposed engagement conceptualization and theoretical model. The study was completed entirely on the web without human interaction to examine engagement *in the wild* with minimal engagement-related confounds [40].

### Participants

To improve external validity, recruitment for the web-based study comprised a national sample of survivors of trauma who had experienced a variety of traumatic events. Specifically, participants were recruited from flyers, social media groups, and the university’s Sona web-based study system. The inclusion criteria were as follows: (1) having experienced at least one traumatic event based on the Diagnostic and Statistical Manual of Mental Disorders, Fifth edition Criterion A [29], (2) English speaking, (3) aged ≥18 years, and (4) having a score >0 on a measure of posttraumatic distress. No other inclusion or exclusion criteria were specified to maximize the number and diversity of respondents.

### Sample Size Criteria

Although most researchers advise a sample size of 10 participants for each parameter being estimated [41], the ratio of sample size to free parameters may be as low as 5:1 with certain model specifications (ie, large factor loading and multiple indicators for each latent variable [42]). This study recruited more participants (at time 1 [T1]; 915/1367, 66.93% who met the criteria) to achieve this minimum while allowing for nonuse, dropout attrition, and flexibility to handle missing data and other unanticipated procedural or methodological issues.

### Materials: DMHI (My Trauma Recovery)

The DMHI used in this study was *My Trauma Recovery* (MTR), which was designed by developers to improve an individual’s CSE-T [43]. MTR is a self-guided, theoretically based, DMHI with no interactions with a therapist. MTR is mainly based on SCT [43] and focuses on increasing an individual’s ability to cope with trauma via six self-directed modules: (1) unhelpful ways of coping, (2) relaxation, (3) social support, (4) self-talk, (5) trauma triggers and memories, and (6) seeking professional help. There is empirical support for the efficacy of MTR, and its mechanism of action (increasing CSE-T) is understood [39].

### Study Design

**Figure 2:**
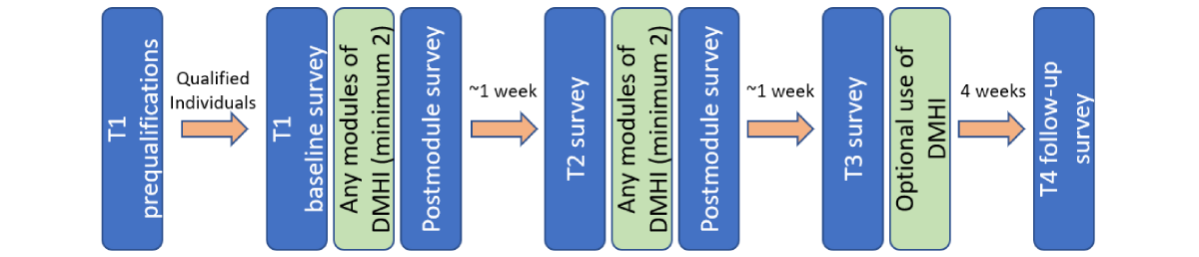
Fully automated, longitudinal research design. The total duration is 6 weeks. DMHI: digital mental health intervention; T1: time 1; T2: time 2; T3: time 3; T4: time 4.

The overall study design is shown in Figure 2. Participants were assigned a random participant identification number to maintain anonymity and track their progress throughout the study. This study used a longitudinal research design comprising 4 periods. T1 was the baseline, T2 was approximately 1 week after T1, and T3 was approximately 2 weeks after T1. Time 4 (T4) was a follow-up questionnaire sent out 30 days after the completion of the study (approximately 6 weeks after T1).

The study was designed to allow participants enough time to practice the skills they learned from the DMHI. Each period indicated the completion of a survey. To move from T1 to T2 and from T2 to T3, the participants had to complete a minimum of 2 modules of the DMHI. Therefore, the individuals moved through the study at different rates. On average, participants moved from one period to the next every 8 days (from T1 to T2: mean 8.88, SD 13.31 days; from T2 to T3: mean 8.49, SD 7.72 days; from T1 to T3: mean M15.67, SD 13.56 days).

After T3, the use of the DMHI was optional. After finishing a module, participants were asked to complete a short, postmodule survey that measured engagement. To increase adherence, automatic reminder emails were sent to participants throughout the study.

### Procedures

#### Overview

Data were collected over 2 years between August 2018 and August 2020. Recruitment followed three primary strategies: (1) Western university’s Sona system posting to recruit undergraduate students, (2) paid advertising on various social media platforms (Facebook, LinkedIn, and Twitter), and (3) free advertising on various email listservs. Sona participants were compensated with extra credit, and web-based participants were compensated US $15 when they completed all study protocols. All participants were entered in a raffle for one of four US $50 gift cards after completing all study protocols (T1, T2, T3, and T4). A list of resources was provided to participants after completing each survey.

#### Screening

All participants were provided with a brief statement explaining the procedure and a link to the study on Qualtrics. Participants indicated that they voluntarily agreed to participate in the web-based informed consent form. After the participants provided informed consent via Qualtrics (by pressing the *I Agree* button), they completed the screening questionnaires. Those who did not meet the criteria (ie, did not experience a traumatic event or were not experiencing any symptoms of posttraumatic distress) were not included in the study.

#### T1 Procedures

Those who met the inclusion criteria were automatically provided the baseline T1 questionnaire, which included all measures assessing demographics, engagement self-efficacy, outcome expectations, and CSE-T. To increase the validity of the responses, 7 questions were embedded throughout the T1 survey as an attention assessment. These questions asked participants to select a specific response (eg, “For this item, please select ‘None at all’”). A valid case was identified as a participant who answered most (4 of 7) validity questions correctly [44].

All participants who validly completed the T1 survey and met the inclusion criteria were provided access to MTR via email. This email provided participants with a link to MTR and instructions on how to create a user account, log into the site, and begin using the site. Participants were asked to watch an introductory video and use MTR as much as needed but were asked to use a minimum of 2 of the 6 modules to receive compensation. To increase adherence, a second reminder email was sent to the participants 3 days after qualification. This email reminded the participants to create an MTR account as soon as possible and contained instructions to do so.

#### Engagement

Objective engagement levels were tracked and recorded by MTR throughout the study. Subjective engagement was assessed after the completion of a module.

#### T2 and T3 Procedures

T2 and T3 used identical procedures and occurred approximately 1 week apart. After completing ≥2 MTR modules, participants were eligible to participate in the next period and were asked to fill out a questionnaire that assessed engagement self-efficacy, activation self-efficacy, CSE-T, and posttraumatic distress. After T3, participants could continue to use MTR as often as needed; however, this was not required.

#### T4 Procedures

A month after completing the T3 survey (approximately 6 weeks from the start of the study), participants received a request via email to fill out a brief T4 questionnaire that assessed skill activation self-efficacy, CSE-T, and posttraumatic distress.

### Measures

The measures used in this study are described in the following sections and are included in Multimedia Appendix 1. The internal consistencies of the measures are included in the *Results* section.

#### Demographics (T1)

Demographic information such as participants’ ethnicity, age, gender, relationship status, income, mental health treatment history, and education was measured.

#### Traumatic Event (T1)

The presence of a Criterion A traumatic event was assessed using the Life Events Checklist-5 (LEC-5), which is a 17-item self-report measure that assesses exposure to potentially traumatic events across the life span [45]. The LEC-5 was used to determine whether participants had experienced a qualifying traumatic event over the course of their lives. If participants endorsed *Happened to me* or *Witnessed it* on the LEC-5, they were eligible for the study. The LEC-5 demonstrated good test-retest reliability (*r*=0.82) [46].

#### Engagement Self-efficacy (T1, T2, and T3)

Engagement self-efficacy was measured using 8 questions at the beginning of T1, T2, and T3. During T1, the questions began with the sentence stem “I am confident that I can begin to use *My Trauma Recovery...*” and during T2 and T3, the same questions began with the sentence stem “I am confident that I can continue to use *My Trauma Recovery...*” Questions comprised items representing technological and coping-related barriers. The answers were provided on a 5-point Likert scale ranging from 1=*not at all confident* to 5=*very confident*.

#### Outcome Expectancies (T1, T2, and T3)

Outcome expectations were measured at the beginning of each period with 9 questions that started with the sentence stem “If I use *MyTraumaRecovery* regularly I expect that*...*” Responses were scored on a 5-point Likert scale ranging from 1=*strongly disagree* to 5=*strongly agree*. Cons were reversed scored, and the total score was computed by summing the answers for all items of the positive and negative outcome expectancies scales.

#### PTSD Symptoms (T1, T2, T3, and T4)

The PTSD checklist for the Diagnostic and Statistical Manual of Mental Disorders, Fifth Edition (PCL-5) [47] was used to measure PTSD symptoms and was anchored to the most relevant trauma on the LEC-5. The PCL-5 was assessed at T1, T2, T3, and the 1-month follow-up. Items assessed symptoms across 4 symptom clusters of PTSD (re-experiencing, avoidance, negative mood, and hyperarousal) on a 0- to 4-point Likert scale. Responses ranged from 0=*not at all* to 4=*extremely*. The PCL-5 was scored using the total symptom severity score (range 0-80) by summing the scores for each of the 20 items.

#### Engagement (T2, T3, and T4)

##### Overview

Engagement was measured both subjectively and objectively. Subjective self-report engagement measures comprised perceived use and attention, interest, and affect. Objective data were automatically measured from the system logs throughout each period. These measures are described in the following sections.

##### Engagement Subjective Use (Postmodule)

The subjective perception of use was measured at the completion of each module and included depth (how much of the module did you use [0%-100%]?) and duration (minutes). In addition, frequency of use was assessed at T2, T3, and T4 with the question “How often did you use *My Trauma Recovery* during the past week?” Answers included 0=*never*, 1=*less than once a week,* 2=*once a week,* 3*=two to three times per week*, 4=*daily*, and 5=*more than once a day.*

##### Engagement Subjective Interest and Attention (Postmodule)

The subjective experiences of interest and attention were measured at the completion of each module. Participants were asked to rank how true several statements were while using the DMHI surrounding interest and attention (eg, “I was absorbed”). The answers were provided on Likert scales, ranging from 0=*not at all true* to 4=*extremely true*.

##### Engagement Subjective Affect (T1 and Postmodule)

The subjective dimension of affect was measured at baseline and after the completion of each module using the Positive and Negative Affect Schedule short form. The Positive and Negative Affect Schedule is a 20-item measure that assesses both positive and negative affect [48]. Each subscale comprised 10 items. The answers were provided on Likert scales, ranging from 1=*not at all* to 5=*extremely*.

##### Engagement Objective Use (Continuous)

Objective engagement measures included continuously recorded data that quantified the frequency (number of logins), breadth (number of pages), depth (number of modules completed), and duration (total number of minutes logged in) of the DMHI use. The data were stored in a secure web-based database.

#### Skill Activation Self-efficacy (T2, T3, and T4)

Skill activation self-efficacy was measured at T2, T3, and T4 using 8 questions that began with the sentence stem “I am confident that I can practice the skills I learned from *My Trauma Recovery...*” followed by items representing coping-related barriers. The answers were provided on a 5-point Likert scale ranging from 1=*not at all confident* to 5=*very confident*.

#### CSE-T (T1, T2, T3, and T4)

CSE-T was assessed at baseline, T2, T3, and T4 using the CSE-T scale [27]. The CSE-T is a 9-item scale that assesses coping self-efficacy for challenges and demands in the trauma recovery process. Questions such as “I feel capable that I can manage distressing dreams or images about the traumatic experience” were measured on a 7-point scale ranging from 1=*not at all capable* to 7=*totally capable*.

### Statistical Analyses

#### Preliminary Analysis

SPSS (version 28; IBM Corp) was used for the demographic and initial analysis. Data were inspected for invalid surveys, outliers, *missingness*, and other characteristics influencing fit before the analyses. The data were then assessed for outliers and normality.

#### Missing Data and Attrition

Missing data were estimated with the full information maximum likelihood procedure using AMOS (version 28; IBM Corp). full information maximum likelihood assumes that data are either missing completely at random (MCAR) or missing at random (MAR) but is also robust when data are missing not at random [49]. The first to be analyzed was the item- or scale-level missing data within each period. Next, tests were performed to analyze missing data because of attrition, which is common in self-directed DMHI longitudinal studies (approximately 99%) [50]. These tests included the Little MCAR test [51], 2-tailed *t* tests, and multinomial regression tests [52].

#### Reliability

SPSS (version 28) was used to calculate reliability. Cronbach α [53] was used to calculate the internal consistency of the measures, where each item was measured on the same scale (ie, all items were measured on a 0-5–point Likert scale). Cronbach α is based on the *essentially tau-equivalent measurement model*, which assumes that each item measures the same latent variable on the same scale (variance) but with possibly different degrees of precision [54]. All measures met this assumption apart from the engagement latent construct, which comprised heterogenous items measured on different scales (eg, pages viewed, interest, affect, and attention) with different SDs. Therefore, the reliability of the engagement latent construct was calculated using the composite reliability (CR) coefficient [55].

#### MTR Effectiveness

Although not the primary focus of this study, outcomes were analyzed with SPSS (version 28) using repeated-measures ANOVAs. Participants used MTR for approximately 2 weeks, during which their PTSD symptoms were measured at baseline (T1), 1-week (T2), 2-week (T3), and 4-week follow-ups (T4; approximately 6 weeks from baseline).

#### Fit Indices

This study evaluated and interpreted model fit on two indices in addition to the chi-square value: (1) comparative fit index (CFI) [42] and (2) root mean square error of approximation (RMSEA) [56]. The guidelines for acceptable fit included a nonsignificant chi-square value. However, it should be noted that the chi-square goodness-of-fit test statistic uses traditional statistical significance testing procedures and is highly subject to the sample size. A CFI >0.90 and RMSEA <0.10 were used as guidelines for acceptable model fit to the data [57]. Given the high attrition rates typically associated with self-directed web-based DMHI studies, more lenient criteria were chosen a priori and were used to evaluate model fit in this study [58].

#### Engagement Measurement Model

To verify the construct validity of the engagement measurement model (hypothesis 1), a confirmatory factor analysis using AMOS (version 28) was used to confirm the factor structure of the set of observed subjective and objective engagement variables. Initially, all observed objective and subjective measures were included, such as objective items measuring the extent of use (minutes, logins, pages, and modules completed) and subjective measures of use, attention, affect, and interest. Items that had a poor factor loading were deleted to improve overall fit unless the deletion compromised the validity of the construct such that it no longer supported engagement conceptualization.

#### Longitudinal Research Model

The proposed longitudinal structural equation model was tested using AMOS (version 28) to confirm hypotheses 2 to 4 (A, B, and C) [59]. Our model specified 2 exogenous predictors of engagement, a multidimensional engagement latent construct, and skill activation self-efficacy and CSE-T as positive serial mediators between engagement and symptom reduction (Figure 1). To analyze the indirect mediational effects between engagement and outcomes, AMOS (version 28) bootstrapping (2000 samples) analysis was performed with bias-corrected CIs (90% CI).

### Ethics Approval

All study materials and procedures were approved by the University of Colorado, Colorado Springs, Institutional Review Board (19-011) before participant contact and data collection.

## Results

### Preliminary Analysis

#### Demographics

Table 1 depicts the descriptive statistics of the demographic variables. Of the 1367 participants who signed up for the study, 915 (66.93%) qualified and completed the T1 survey. The participants who met the criteria were 76.9% (704/915) White, 17.8% (163/915) Hispanic or Latino, 7.4% (68/915) Black, 6.4% (59/915) Asian or Pacific Islander, 4.7% (43/915) Native American or Alaskan Native, and 3.3% (30/915) other (some participants specified multiple races). Of the 915 who qualified, 404 (44.1%) created an account on the DMHI, 350 (38.2%) participated in the T2 survey, and 168 (18.4%) participated in T3. Of the 168 participants who completed the T3 survey, 101 (60.12%) completed the T4 1-month follow-up survey.

All participants who met the criteria were directly exposed to ≥1 traumatic event either through experiencing or witnessing the event, including an accident (741/915, 81%), physical assault (564/915, 61.6%), sexual assault (547/915, 59.8%), life-threatening illness or injury (472/915, 51.6%), natural disasters (370/915, 40.4%), sudden unexpected death of someone close (350/915, 38.3%), military combat (72/915, 7.9%), captivity (51/915, 5.6%), and other distress (588/915, 64.3%).

Data were assessed for outliers and normality. A series of comparisons using ANOVA (Cronbach α=.05) were conducted to determine whether any relevant differences existed in the variables of interest (eg, engagement variables, activation self-efficacy, and CSE-T) by demographic characteristics (eg, age and education). No significant differences were found. Regarding multicollinearity, there was no correlation between variables above >0.80. Therefore, there was no indication of collinearity [60].

**Table 1:** Descriptive statistics for demographics for time 1, time 2, time 3, and time 4 (N=915)^a^.

Measure		Time 1 (N=915)	Time 2 (n=350)	Time 3 (n=168)	Time 4 (n=101)
Age (years), mean (SD; range)		24.11 (8.53; 18-62)	26.13 (9.39; 18-60)	28.12 (9.04; 18-60)	30.32 (9.08; 18-54)
**Gender, n (%)**					
	Female	698 (76.3)	270 (77.1)	117 (69.7)	67 (66.3)
	Male	205 (22.4)	77 (22.0)	49 (28.9)	34 (33.7)
	Other	10 (1.1)	3 (1)	2 (1.1)	0 (0)
**Relationship status, n (%)**					
	Single	646 (70.6)	219 (62.6)	96 (57)	53 (52.4)
	Married	143 (15.6)	73 (20.9)	46 (27.5)	34 (33.7)
	Divorced	48 (5.2)	27 (7.7)	14 (8.4)	10 (9.9)
	Widowed	5 (0.5)	2 (0.6)	1 (0.6)	1 (1)
	Other	73 (8)	29 (8.3)	11 (6.5)	3 (3)
**Highest education, n (%)**					
	High School	173 (18.9)	57 (16.3)	26 (15.5)	14 (14.1)
	Some college	617 (67.4)	225 (64.3)	98 (58.4)	52 (51.5)
	Bachelor’s degree	81 (8.9)	45 (12.9)	31 (18.3)	24 (23.9)
	Graduate degree	35 (3.8)	20 (5.7)	13 (7.7)	11 (10.8)
Sona (vs web-based), n (%)		713 (77.9)	238 (68)	83 (49.3)	29 (28.3)
**Mental health**					
	Treatment (current), n (%)	248 (27.1)	117 (33.5)	62 (36.7)	33 (32.6)
	Treatment (lifetime), n (%)	548 (59.9)	246 (70.3)	114 (67.8)	72 (71.7)
	Baseline PCL-5^b^, mean (SD)	35.83 (19.10)	40.14 (18.27)	41.98 (18.32)	44.99 (16.97)
**Traumatic event, mean (SD)**					
	Frequency (lifetime)	9.79 (19.01)	10.49 (15.63)	11.36 (16.32)	10.92 (16.46)
	Intensity (0-5)	3.08 (0.93)	3.27 (0.79)	3.95 (0.97)	4.12 (0.91)

^a^Some percentages did not add up to 100% because of missing data.

^b^PCL-5: PTSD checklist for the Diagnostic and Statistical Manual of Mental Disorders, Fifth Edition.

#### Missing Data and Attrition

The missing data for all relevant items within each period were 0.012% for T1, 0.006% for T2, and 0.005% for T3. As all item-level missing data per period were <1%, they were deemed negligible.

Of the 915 participants who met the criteria for the study, 511 (55.8%) did not create an account (ie, nonuse attrition). Of the 404 participants who created an account, 236 (58.4%) did not complete the T3 protocol. Hence, the nonuse and dropout rates were 55.8% and 58.4%, respectively. To analyze the missing data patterns, the Little MCAR test, a stricter criterion than MAR, was performed with all variables used in the full model (T1, T2, and T3) simultaneously, with age as a reference variable. The results of this test showed that the missing data were not MCAR (*χ^2^*_34_= 278.2; *P<*.001). This suggests that missing data from T1 to T3 are either MAR or missing not at random; however, there are no definitive tests for these conditions [61].

Independent-sample 2-tailed *t* tests (equal variances assumed) showed significant baseline differences between noncompleters and completers on the predictors of engagement. Completers reported higher engagement self-efficacy (noncompleters: mean 16.85, SD 7.40; completers: mean 20.71, SD 6.70; *t*_915_=−6.21; *P<*.001) and higher outcome expectations (noncompleters: mean 21.91, SD 5.21; completers: mean 24.23, SD 4.61; *t*_915_=−5.33; and *P<*.001).

Multinomial logistic regression was used to identify covariates and interactions that were simultaneously predictive of missingness for the different groups (ie, nonuse, dropouts, and completers). This allows researchers to reasonably assume that the data are MAR. All baseline measures were included (eg, age, education, baseline symptoms, engagement self-efficacy, and outcome expectations), and the results indicated that engagement self-efficacy (*B*=−0.07; odds ratio=0.93; *P=*.003; 95% CI 0.91-0.97) and outcome expectations (*B*=−0.11; odds ratio=0.90; *P=*.01, 95% CI 0.88-0.95) were the only significant predictors of dropout or nonuse group membership. As these significant covariates were included in the model, bias because of missingness may be reduced, and the assumptions of maximum likelihood were assumed to be met.

#### Reliability

Cronbach α [53] or the CR coefficient [54] was calculated for each measure in the model. The results are shown in Table 2.

**Table 2:** Internal reliability for T1^a^ (N=915), T2^b^ (n=350), T3^c^ (n=168), and T4^d^ (n=101) measures.

Scale			Number of items		Cronbach α						
					T1		T2		T3		T4
**Engagement predictors**											
	Engagement self-efficacy	8		.94		.95		.95		—^e^	
	Outcome expectations	9		.84		.86		.78		—	
**Engagement variables and latent construct**											
	Attention or interest	7		—		.86		.84		.81	
	PANAS^f^ (positive affect)	10		.89		.94		.93		.94	
	Subjective frequency	6		—		.89		.91		.96	
	Engagement latent construct (*composite reliability*)	4		—		.70		.66		.74	
**Outcome predictors**											
	Activation self-efficacy	8		—		.97		.96		.97	
	Trauma coping self-efficacy	9		.91		.92		.93		.93	
**Outcome**											
	PCL-5^g^	20		.95		.96		.97		.98	

^a^T1: time 1.

^b^T2: time 2.

^c^T3: time 3.

^d^T1: time 4.

^e^Indicates scale not measured during the period.

^f^PANAS: Positive and Negative Affect Schedule.

^g^PCL-5: PTSD checklist for the Diagnostic and Statistical Manual of Mental Disorders, Fifth Edition.

### MTR Effectiveness

The average baseline PTSD symptom severity, as indicated by the PCL-5, was above the 33.00 cutoff value suggested by the National Center for PTSD (T1: mean43.77, SD 17.43). A repeated-measures ANOVA showed that PTSD symptoms differed significantly between time points (Wilks λ=0.507; *F*_3,97_=31.42; *P<*.001), with a large effect size (η^2^=0.49). Post hoc tests using the Bonferroni correction revealed that PTSD was reduced by an average of 11.10 points on the PCL-5 after 1 week (*P<*.001), 13.25 after 2 weeks (*P<*.001), and 18.15 (*P<*.001) after 6 weeks.

### Engagement Measurement Model

**Figure 3:**
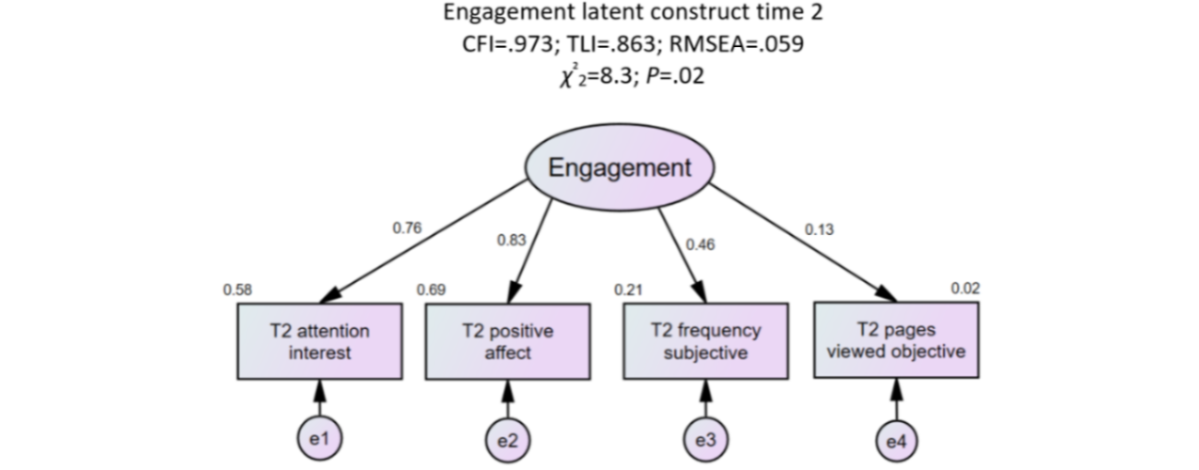
Engagement latent confirmatory factor analysis that includes attention, interest, affect, subjective frequency of use, and objective pages viewed. Results of the confirmatory factor analysis provided an adequate model fit and reliability. All regression weights were significant, *P*<.05. CFI: comparative fit index; RMSEA: root mean square error of approximation; T2: time 2; TLI: Tucker-Lewis index.

The initial test of the engagement latent construct provided a poor fit to the data (*χ^2^*_27_=347.8; *P<*.001; CFI 0.663; RMSEA 0.134; 90% CI 0.122-0.146). Nonsignificant and poor loading factors were deleted to improve the model fit. The final engagement measurement model comprised attention or interest (β=.76; *P<*.001), positive affect (β=.83; *P<*.001), subjective measure of frequency (β=.46, *P<*.001), and the objective measure of pages viewed (β=.13, *P=*.02). The excellent fit (*χ^2^*_2_=8.3; *P=*.02; CFI 0.973; RMSEA 0.059; 90% CI 0.022-0.103) of the confirmed engagement model supported hypothesis 1 (Figure 3) and was consistent with the engagement definition [15].

### Longitudinal Research Model

#### Overview

**Figure 4:**
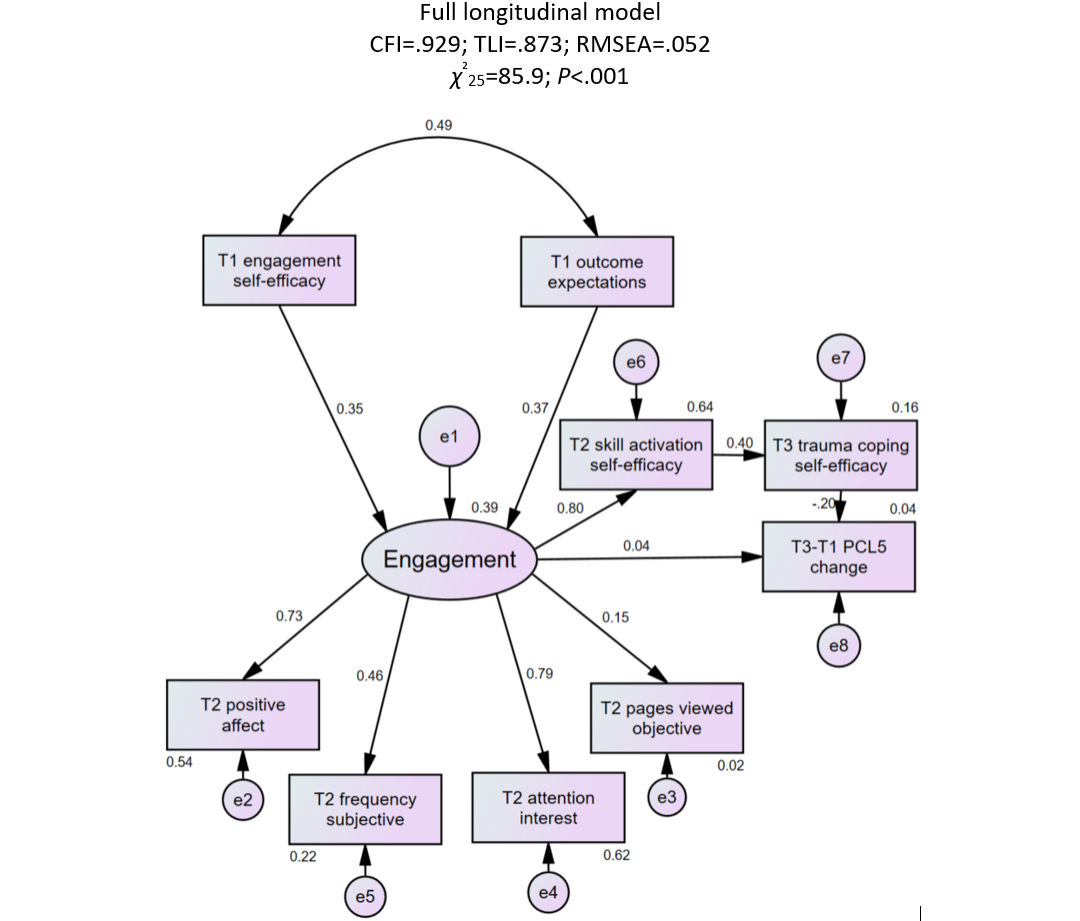
Full longitudinal structural equation modeling results where engagement self-efficacy and outcome expectations were significant predictors of engagement. The direct effect of engagement on symptom improvement was nonsignificant. The indirect serial mediation pathway between engagement and symptom improvement was significant in that engagement predicted increases in skill activation self-efficacy, which then mediated subsequent increases in coping self-efficacy for trauma and reductions in posttraumatic stress disorder symptoms. CFI: comparative fit index; PCL-5: Posttraumatic Stress Disorder checklist for Diagnostic and Statistical Manual of Mental Disorders, Fifth Edition; RMSEA: root mean square error of approximation; T1: time 1; T2: time 2; T3: time 3; TLI: Tucker-Lewis index.

The engagement latent construct was used in the longitudinal research model (Figure 1). Table 3 shows the bivariate correlation coefficients for the study variables included in the tested model. Overall, the model demonstrated good fit (*χ*^²^_25_=85.9; *P<*.001; CFI 0.929; RMSEA 0.052; 90% CI 0.040-0.064) and supported hypotheses 2 to 4 (Figure 4). The details of this process are described below.

**Table 3:** Correlations of variables used in the full structural equation model: T1^a^ (N=915), T2^b^ (n=350), and T3^c^ (n=168)^d,e^.

Categories		Predictors			Engagement					Outcomes		
		1	2	3		4	5	6	7		8	9
**Variables**												
	T1 engagement self-efficacy	1	0.489^f^	0.328^f^		0.296^f^	0.356^f^	0.195^f^	0.552^f^		0.089	−0.094
	T1 outcome expectations	—^g^	1	0.362^f^		0.375^f^	0.330^f^	0.112^g^	0.476^f^		0.093	−0.121^g^
	T2 subjective affect positive	—	—	1		0.574^f^	0.385^f^	0.069	0.538^f^		0.150	0.038
	T2 subjective interest or attention	—	—	—		1	0.443^f^	0.077	0.577^f^		0.145	0.001
	T2 subjective engagement frequency	—	—	—		—	1	0.062	0.404^f^		−0.068	−0.026
	Objective engagement pages viewed	—	—	—		—	—	1	0.122^h^		0.037	−0.153^e^
	T2 skill activation self-efficacy	—	—	—		—	—	—	1		0.368^h^	−0.032
	T3 CSE-T^i^	—	—	—		—	—	—	—		1	−0.189^h^
	T3 to T1 PCL-5^j^	—	—	—		—	—	—	—		—	1

^a^T1: time 1.

^b^T2: time 2.

^c^T3: time 3.

^d^Objective measures were continuously measured.

^e^Predictor 1: mean 17.56 (SD 7.43); predictor 2: mean 22.33 (SD 5.18); engagement 3: mean 10.79 (SD 7.14); engagement 4: mean 14.83 (SD 4.45); engagement 5: mean 2.36 (SD 1.07); engagement 6: mean 84.69 (SD 63.42); outcomes 7: mean 26.65 (SD 6.98); outcomes 8: mean 42.52 (SD 10.89); outcomes 9: mean −10.78 (SD 15.86).

^f^*P<*.01.

^g^Not applicable.

^h^*P<*.05.

^i^CSE-T: coping self-efficacy for trauma.

^j^PCL-5: PTSD checklist for the Diagnostic and Statistical Manual of Mental Disorders, Fifth Edition.

#### Engagement Predictors

Analysis of the hypothesized predictors of engagement indicated that engagement self-efficacy (β=.35; *P<*.001) and outcome expectations (β=.37; *P<*.001) were significant positive predictors of engagement (*R*^2^=39%). Adequate fit and significant pathways supported hypotheses 2 to 3.

#### Engagement Outcomes

Regarding the relationship between engagement and outcomes, the direct effect of engagement on changes in PTSD symptoms was nonsignificant (β=.04; *P=*.58). However, the indirect serial mediational pathway between engagement and T3 to T1 PTSD symptom reduction was found to be statistically significant (β=−.065; *P<*.001; 90% CI −0.071 to −0.058]). Examination of the mediators between engagement and symptom change revealed that engagement was a strong and significant predictor of T2 skill activation self-efficacy (β=.80; *P<*.001; *R*^2^=64%), and skill activation self-efficacy was a significant predictor of T3 CSE-T (β=.40; *P<*.001; *R*^2^=16%). Importantly, the CSE-T significantly predicted PTSD symptom reduction between T1 and T3 (β=−.20; *P*=.02; *R*^2^=4%). Thus, these results provided support for hypotheses 4A, 4B, and 4C (Figure 4).

#### Exploratory Model

In addition to the above model, the second similar model included those who completed the 4-week follow-up (101/915, 11%). The T1 to T4 model provided an excellent fit (*χ^2^*_25_=49.8; *P=*.002; CFI 0.950; RMSEA 0.033; 90% CI 0.019-0.046), suggesting that at the 4-week follow-up, participants continued to show significant improvement through the indirect effects of skill activation self-efficacy and CSE-T. Combined, the above results support hypotheses 1 to 4.

## Discussion

### Principal Findings

The present research aimed to address the gap in the engagement literature surrounding the definition, measurement, and modeling of engagement with the ultimate goal of understanding ways of effectively increasing engagement. Using a multidimensional definition of engagement that included both subjective and objective components, the proposed conceptualization was tested with a trauma recovery DMHI. As far as we are aware, this is the first study that offers empirical support for a multidimensional definition of engagement. On the basis of the confirmed measurement model of engagement, a theoretically based model of DMHI engagement was tested with a national sample of survivors of trauma. The results confirmed the validity and reliability of the comprehensive engagement measurement model and the relationships between the DMHI engagement, predictors of engagement, and clinical outcomes.

A strength of this study is the variety of trauma-exposed individuals recruited. Trauma experiences included accidents, physical and sexual assault, natural disasters, and military combat. On average, survivors of trauma reported baseline PTSD scores that may be interpreted as above the diagnostic threshold (mean 35.83, SD 19.10), and those who completed the study experienced, on average, a clinically significant reduction in PTSD symptoms (mean 13.24 point reduction on the PCL-5, SE 1.83; *P*<.001; n=168)) that persisted at the 1-month follow-up (mean 18.15-point reduction on the PCL-5, SE 1.86; *P*<.001; n=101).

### Engagement Measurement Model

The final model demonstrated adequate reliability in this sample (CR=0.70) and included all required components of the proposed definition. A strength of this model is that it is not DMHI specific; rather, it contains general measures of attention, interest, affect, and use that could be applied to other applications, although this has yet to be determined. Another advantage is its parsimony. Measuring subjective experiences while using a DMHI can be burdensome for users [62]. Therefore, short, valid, and reliable measures of engagement may increase compliance. Importantly, the final model did not confound the predictors of engagement (eg, aesthetics and satisfaction) with engagement.

### Longitudinal Research Model

#### Engagement Predictors

The results revealed that 2 exogenous variables, engagement self-efficacy (β=.35; *P<*.001) and outcome expectations (β=.37; *P<*.001), were significant predictors of engagement. This confirms previous research, where engagement self-efficacy and outcome expectancies were major determinants of DMHI use [17,63,64]. In these studies, highly motivated participants felt capable of using the DMHI and perceived it as useful and worth the effort.

#### Engagement Outcomes

The relationship between engagement and outcomes is not well understood [14]. Our model tested 2 different task-specific self-efficacies as serial mediators between engagement and outcomes. Specifically, engagement was found to influence skill activation self-efficacy, where those with higher levels of engagement experienced greater levels of skill activation self-efficacy (ie, belief in the ability to enact skills learned through the DMHI). In turn, higher levels of skill activation self-efficacy predicted higher levels of CSE-T, which mediated an improvement in PTSD symptoms.

Skill activation self-efficacy has been shown to increase health management behaviors in nondigital health care settings, such as heart failure [65], diabetes management [66], and HIV [67], but, as far as we are aware, has never been tested as a DMHI mechanism of action. As predicted by SCT, our results suggest that augmenting beliefs about personal efficacy in DMHI skills practice may be an antecedent to improved confidence in managing posttrauma recovery demands.

Similar to previous research, our study found that CSE-T was the most proximal predictor of symptom improvement. This confirms other studies in which CSE-T mediated posttrauma recovery from several traumatic experiences [68], including accidents [69], sexual abuse [70], life-threatening illnesses [71], and natural disasters [72]. Combined, skill activation and CSE-T mediated the relationship between DMHI engagement and outcomes. This finding is consistent with an extensive literature base that identifies cognitive changes as mediators of mental health symptom improvement (refer to Ehlers et al [73] and Kleim et al [74]). However, the relatively small amount of explained outcome variance (*R*^2^=4%) suggests that there may be additional mechanisms of action not included in the model.

Interestingly, the direct pathway between engagement and symptom reduction was not significant after 2 weeks. This supports previous short-term research that failed to find a relationship between engagement and outcomes [75-77] and underscores the importance of understanding and targeting the mechanisms of action between engagement and outcomes to improve DMHI efficacy. Simply increasing engagement to improve outcomes without considering these mediating factors may not suffice.

### Limitations

#### Overview

A major limitation of this model is that it views predictors, engagement, and outcomes as unidirectional processes in which predictors influence engagement and engagement influences the outcomes. According to SCT, behaviors, personal factors, and the environment interact with each other over time (ie, triadic reciprocal causation). It is highly probable that these components operate in a nonrecursive fashion of reciprocal determinism. Further examination of this dynamic framework can reveal how engagement changes and influences predictors and recovery as it unfolds across time. Modeling these dynamic reciprocating processes is beyond the scope of this study and will be investigated in future studies.

#### Attrition

This study had a high attrition rate, which is consistent with other longitudinal DMHI studies [78,79]. Attrition can cause potential biases and threats to generalizability [51].

#### Engagement Latent Construct

Several limitations surround the engagement latent construct. As seen in much of the literature, different objective measures have been used to define engagement with disparate results [14,79]. For this study, only 1 objective measure was included in the final model. Researchers have suggested that multiple objective and subjective measures may more accurately represent engagement [80]. However, the equivocal findings in the DMHI engagement literature suggest that the most appropriate measure of use may vary for each DMHI. Further improvements to these measures may be warranted. Although the final model had an excellent fit and supported hypothesis 1, the relatively weak factor loading and explained variance leave some questions for discussion and future research regarding which components are most relevant.

In addition, SCT suggests that predictors and outcomes of engagement influence engagement throughout the DMHI experience [20]. Future research may want to examine the differential and recursive effects of outcomes such as CSE-T and symptom reduction on engagement. An examination of engagement over time may reveal that other measures of engagement may become more influential as users move deeper into the intervention and experience greater (or lesser) changes because of their efforts [81].

#### Study Design

This study used a longitudinal correlational design, suggesting that cause and effect are only interpreted based on theory and time lag and not on experimental manipulation. The fitted models do not necessarily represent the only true models, and there may be others that also fit the data [41]. Several engagement predictors were not investigated in this study, such as social support [64]. This will be an area for future exploration.

Regarding outcomes, skill activation self-efficacy is assumed to increase the practice of DHMI skills. However, a measure of skill practice was not included in our model. Ideally, an accurate measure of skill practice should be captured through daily ecological momentary assessments. Future studies should incorporate daily ecological momentary assessments of skill use as a mediator in symptom reduction.

#### DMHI-Related Limitations

In this study, only 1 DMHI was tested that targeted a mental health disorder (PTSD). MTR is a web-based web intervention that does not use many recent advances in digital technology such as social networking, virtual reality, machine learning, sensor technology, and mobile computing. Examination of the engagement measurement model and theoretical models with flexible and novel DMHIs for a variety of mental health issues may help confirm the generalizability of these findings.

Due to the design of MTR, participants were led through each module by way of several predetermined steps (ie, tunneling). The participants generally moved through the intervention at the same rate, which provided limited variability in engagement use patterns. These types of tunneled interventions have been found to generate more page views than self-paced interventions [79]. However, this may be an artifact of making users click through a prespecified number of pages to progress through the DMHI and may not be at all related to engagement.

#### Sample

Although a national sample of survivors of trauma was recruited from throughout the United States, most of the participants were White female psychology students enrolled in a Western university.

### Implications

#### Engagement

The findings of this study established the validity and reliability of a multidimensional engagement measurement model, although questions for future research remain. In principle, empirically supported behavioral and experiential dimensions of engagement can be measured in every DMHI. With a valid and reliable measurement of engagement, the therapeutic dose of DMHIs can be established, and the relationships between individual characteristics, engagement, and intervention effectiveness can be better understood. Ultimately, an adequate measure of engagement may provide the opportunity to automatically detect disengagement and help identify the factors that improve engagement.

#### Theoretical

This study provides a theoretical foundation for understanding numerous predictors of engagement. Although several models could potentially fit the data, the present findings tend to replicate earlier findings in the context of engagement predictors and are in line with the SCT.

#### Clinical

Importantly, these findings have implications for mental health interventions, whether in person or on the web. Treatment dropout and its causes remain top research priorities in both settings [82]. Improving engagement can potentially lead to improved therapeutic outcomes. By understanding the impact of engagement self-efficacy and outcome expectations, interventions can be designed to enhance these perceptions before treatment, which could, in turn, lead to improved engagement. Skill activation self-efficacy and the CSE-T were shown to mediate the path from engagement to symptom reduction. Although skill training is an essential component of most DMHIs, ensuring that users feel confident in practicing those skills appears to be an important component of DMHI effectiveness.

### Directions for Future Research

This research provides a strong foundation for several different explorations surrounding DMHI engagement. Although subjective measures demonstrated strong factor loadings in the engagement measurement model, low response rates to embedded DMHI engagement surveys were common. Combining nonintrusive sensor data with machine learning may be an important area of research to help alleviate the participant burden [83].

This study also provides support for future research on engagement predictors. We offered a theoretically based predictor model. Future experiments that manipulate predictors of engagement, such as outcome expectations, are encouraged.

Logically, engagement alone does not make an intervention effective. Our model revealed the significant indirect effects of engagement on symptom reduction. A further understanding of the mechanisms of action may contribute to overall intervention effectiveness [84]. In theory, these components can be a part of every DMHI.

### Conclusions

The empirically supported engagement latent construct and structural equation model provide steps toward formalizing the science of engagement. In turn, this may help improve the design of engaging and effective digital interventions. Unique individual difference variables related to engagement may then emerge, offering a more refined approach to intervention customization.

The therapeutic dose of DMHIs can be established with a valid and reliable measurement of engagement, and DMHI efficacy can be evaluated in a more standardized way. Comparisons among similar DMHIs can then be accomplished through clinical trials to establish the safety and effectiveness of the DMHI. Once established, DMHIs can be designed to increase engagement in early interventions, meet the specific needs of populations, and be used at the exact moment they are needed. Taken together, the future is bright for the role of DMHIs in overcoming significant barriers to care and improving outcomes for a variety of mental health disorders.

## Appendix

Multimedia Appendix 1 Measures used in this research study.

## Abbreviations

CFI: comparative fit index
CR: composite reliability
CSE-T: coping self-efficacy for trauma
DMHI: digital mental health intervention
LEC-5: Life Events Checklist-5
MAR: missing at random
MCAR: missing completely at random
MTR: My Trauma Recovery
PCL-5: PTSD checklist for Diagnostic and Statistical Manual of Mental Disorders, Fifth Edition
PTSD: posttraumatic stress disorder
RMSEA: root mean square error of approximation
SCT: social cognitive theory
T1: time 1
T2: time 2
T3: time 3
T4: time 4
