# Supplementary material for: Engagement, Predictors, and Outcomes of a Trauma Recovery Digital Mental Health Intervention: Longitudinal Study
Source: JMIR Ment Health. 2022 May 2;9(5):e35048. doi: 10.2196/35048 (PMC9112079; doi:10.2196/35048)
Supplement: Multimedia Appendix 1 [file mental_v9i5e35048_app1.docx]

# Measures

## Screening - Traumatic Event (LEC-5)

Listed below are several difficult or stressful things that sometimes happen to people. For each event, check one or more of the boxes to the right to indicate that: (a) it happened to you personally; (b) you witnessed it happen to someone else; (c) you learned about it happening to a close family member or close friend; (d) you were exposed to it as part of your job (for example, paramedic, police, military, or other first responder); (e) you’re not sure if it fits; or (f ) it doesn’t apply to you.

Be sure to consider your entire life (growing up as well as adulthood) as you go through the list of events.

| 1 | 2 | 3 | 4 | 5 | 6 |
| --- | --- | --- | --- | --- | --- |
| Happened to me | Witnessed it | Learned about it | Part of job | Not sure | Doesn’t apply |

1. Natural disaster (for example, flood, hurricane, tornado, earthquake)
2. Fire or explosion
3. Transportation accident (for example, car accident, boat accident, train wreck, plane crash)
4. Serious accident at work, home, or during recreational activity
5. Exposure to toxic substance (for example, dangerous chemicals, radiation)
6. Physical assault (for example, being attacked, hit, slapped, kicked, beaten up)
7. Assault with a weapon (for example, being shot, stabbed, threatened with a knife, gun, bomb)
8. Sexual assault (rape, attempted rape, made to perform any type of sexual act through force or threat of harm)
9. Other unwanted or uncomfortable sexual experience
10. Combat or exposure to a war-zone (in the military or as a civilian)
11. Captivity (for example, being kidnapped, abducted, held hostage, prisoner of war)
12. Life-threatening illness or injury
13. Severe human suffering
14. Sudden violent death (for example, homicide, suicide)
15. Sudden accidental death
16. Serious injury, harm, or death you caused to someone else
17. Any other very stressful event or experience

## PTSD (PCL-5)

Below is a list of problems and complaints that people sometimes have in response to stressful life experiences. With reference to what you selected in the previous question, read each of the problems and then indicate how much you have been bothered by that problem in the **past week.**

| 0 | 1 | 2 | 3 | 4 |
| --- | --- | --- | --- | --- |
| Not at all | A little bit | Moderately | Quite a bit | Extremely |

1. Repeated, disturbing, and unwanted memories of the stressful experience?
2. Repeated, disturbing dreams of the stressful experience?
3. Suddenly feeling or acting as if the stressful experience were actually happening again (as if you were actually back there reliving it)?
4. Feeling very upset when something reminded you of the stressful experience?
5. Having strong physical reactions when something reminded you of the stressful experience (for example, heart pounding, trouble breathing, sweating)?
6. Avoiding memories, thoughts, or feelings related to the stressful experience?
7. Avoiding external reminders of the stressful experience (for example, people, places, conversations, activities, objects, or situations)?
8. Trouble remembering important parts of the stressful experience.
9. Having strong negative beliefs about yourself, other people, or the world (for example, having thoughts such as: I am bad, there is something seriously wrong with me, no one can be trusted, the world is completely dangerous)?
10. Blaming yourself or someone else for the stressful experience or what happened after it?
11. Having strong negative feelings such as fear, horror, anger, guilt, or shame?
12. Loss of interest in activities that you used to enjoy.
13. Feeling distant or cut off from other people?
14. Trouble experiencing positive feelings (for example, being unable to feel happiness or have loving feelings for people close to you)?
15. Irritable behavior, angry outbursts, or acting aggressively?
16. Taking too many risks or doing things that could cause you harm?
17. Being “superalert” or watchful or on guard?
18. Feeling jumpy or easily startled?
19. Having difficulty concentrating?
20. Trouble falling or staying asleep?

## Engagement Self-Efficacy

| 0 | 1 | 2 | 3 | 4 |
| --- | --- | --- | --- | --- |
| Not at all confident | Slightly confident | Somewhat confident | Moderately confident | Very confident |

For each statement described below, please rate how confident you are.

I am confident I can use *My Trauma Recovery* ...

1. Even if I cannot see any positive changes immediately.
2. Even if I did not initially like it.
3. Even if it takes me a long time to learn how to use it.
4. Even if I do not feel relaxed.
5. Even if it brings up difficult memories.
6. Even if I have difficulty applying the things I learn to my situation.
7. Even if it makes me uncomfortable.
8. Even if I have difficulty finding social support.

## Outcome Expectations

If I used ***My Trauma Recovery*** on a regular basis I expect that …

| 0 | 1 | 2 | 3 | 4 |
| --- | --- | --- | --- | --- |
| Strongly disagree | Disagree | Neither Agree or Disagree | Agree | Strongly agree |

1. I will feel more balanced in my daily life.
2. It will help me to relax more.
3. It will take too much of my time.
4. I will be less vulnerable to anxiety and or depression.
5. It will not make any difference in how I feel.
6. I will be able to take better care of myself.
7. I will be able to get more positive support from people in my life.
8. It will make me feel worse.
9. I will feel more in control of my life.

## Engagement - Subjective Experience - Interest/Attention

Please complete the items below by indicating the choice that best describes your overall experiences and reactions while you were working on the website module.

| 0 | 1 | 2 | 3 | 4 |
| --- | --- | --- | --- | --- |
| Not at all true | Slightly true | Somewhat true | Very true | Extremely true |

1. I was interested.
2. I was bored.
3. I was captivated.
4. I was inattentive.
5. I was absorbed.
6. I was detached.

## Engagement - Subjective Usage

Please indicate how much of the website module you used?

Slider bar from 0-100 [None at all – All of the module]

Please estimate how many minutes you spent on the website module you just completed.

[ ]

## Engagement – Subjective Experience - Affect/Mood Positive and Negative Affect Schedule (PANAS-SF)

Please describe how you feel right now by indicating the most appropriate choice after each of the sentences listed below.

| 1 | 2 | 3 | 4 | 5 |
| --- | --- | --- | --- | --- |
| Not at all | A little bit | Moderately | Quite a bit | Extremely |

- Happy
- Irritable
- Distressed
- Alert
- Excited
- Ashamed
- Upset
- Inspired
- Strong
- Nervous
- Guilty
- Determined
- Scared
- Empowered
- Hostile
- Jittery
- Enthusiastic
- Numb
- Proud
- Afraid

## Activation Self-Efficacy

Please indicate confident you are that you can practice the skills you learned from *My Trauma Recovery* in the event that any of the following circumstances were to occur

| 0 | 1 | 2 | 3 | 4 |
| --- | --- | --- | --- | --- |
| Not at all confident | Slightly confident | Somewhat confident | Moderately confident | Very confident |

I am confident I can practice the skills I learned from *My Trauma Recovery* ...

- Even if I have limited time.
- Even if it is difficult to do.
- Even if I have trouble learning how to do them.
- Even if it looks like they are not working initially.
- Even if I feel nervous and stressed.
- Even if I have trouble coping with important changes in my life.
- Even if I have difficulty handling the things I have to do.
- Even if I am having personal problems.

## Demographics

What is your gender?

| 1 | 2 | 3 |
| --- | --- | --- |
| Male | Female | Other |

What is your sex?

| 1 | 2 | 3 |
| --- | --- | --- |
| Male | Female | Other |

What is your date of birth? (MM/YYYY)

What is your current relationship status?

| 1 | 2 | 3 | 4 | 5 | 6 | 7 |
| --- | --- | --- | --- | --- | --- | --- |
| Single (never married) | Married | With Partner | Separated | Divorced | Widowed | Other |

If you answered “Other” to the question above, please specify.

[ ]

What is your ethnicity? (Select all that apply)

| 1 | 2 | 3 | 4 | 5 | 6 | 7 |
| --- | --- | --- | --- | --- | --- | --- |
| Native American or Alaskan Native | Asian or Pacific Islander | Black or African American | Hispanic or Latino | White/ Caucasian | Prefer not to answer | Other |

If you answered “Other” to the question above, please specify.

[ ]

What is the highest level of school you have completed or the highest degree you have received?

| 1 | 2 | 3 | 4 | 5 | 6 | 7 |
| --- | --- | --- | --- | --- | --- | --- |
| High school diploma | Some college work | Associate degree | Bachelor’s degree | Master’s degree | Doctorate or professional degree | Other |

If you answered “Other” to the question above, please specify.

[ ]

What is your annual household income?

[ ]

Are you currently seeing a mental health provider (therapist, counselor, psychiatrist)?

| 1 | 2 |
| --- | --- |
| Yes | No |

Have you seen a mental health provider in the past?

| 1 | 2 |
| --- | --- |
| Yes | No |

If you answer "Yes" to the previous question, please specify how long ago you saw a mental health provider.

[ ]
